# Alpha-tubulin acetyltransferase/MEC-17 regulates cancer cell migration and invasion through epithelial–mesenchymal transition suppression and cell polarity disruption

**DOI:** 10.1038/s41598-018-35392-6

**Published:** 2018-11-30

**Authors:** Cheng-Che Lee, Yun-Ching Cheng, Chi-Yen Chang, Chi-Min Lin, Jang-Yang Chang

**Affiliations:** 1 Center of Infectious Disease and Signaling Research, College of Medicine, National Cheng Kung University, Tainan, ROC Taiwan; 2 Department of Medical Research, Chang Bing Show Chwan Memorial Hospital, Changhua, ROC Taiwan; 3 National Institute of Cancer Research, National Health Research Institutes, Tainan, ROC Taiwan; 4 Division of Hematology and Oncology, Department of Internal Medicine, National Cheng Kung University Hospital, College of Medicine, National Cheng Kung University, Tainan, ROC Taiwan

## Abstract

MEC-17, a newly identified alpha-tubulin-N-acetyltransferase 1, serves as the major α-tubulin acetyltransferase to promote α-tubulin acetylation *in vitro* and *in vivo*. Alteration of α-tubulin acetylation may be involved in morphology regulation, cell migration, and tumour metastasis. However, MEC-17’s role in cell physiology and its effect on epithelial–mesenchymal transition (EMT) and cell polarity remain elusive. In the present study, we characterized the overexpressed or downregulated cell models through gene targeting as MEC-17 gain- or loss-of-function. Overexpression of MEC-17 enhanced the cell spreading area, suppressed pseudopods formation in a three-dimensional (3D) culture system, and inhibited cancer cell migratory and invasive ability and tumour metastasis by orthotopic lung cancer animal model. Furthermore, morphological change and migration inhibition of cancer cells were accompanied by EMT repression, Golgi reorientation, and polarity disruption caused by alteration of cdc42 activity via a decrease in Rho-GAP, ARHGAP21. By contrast, a reduction in endogenous MEC-17 accelerated the pseudopods formation and EMT, and facilitated cell migration and invasion. These results demonstrated the crucial role of MEC-17 in the modulation of intrinsic cell morphogenesis, migration, and invasive function through regulation of EMT and cell polarity.

## Introduction

Epithelial–mesenchymal transition (EMT) is a crucial process that causes epithelial cells to acquire mesenchymal-like morphology and properties, thereby increasing their migration ability^1,2^. EMT plays an essential role during embryogenesis^3,4^ and organ development, and its abnormal pathological activation during cancer progression transforms primary tumours into invasive metastatic ones^5–7^.

Posttranslational modifications (PTMs), including acetylation, detyrosination, polyglutamination and polyglycylation, cause eukaryotic cells to generate distinct microtubule subtypes^8^. In particular, aberrant acetylation of nonhistone proteins results in a variety of cellular actions involved in critical biological regulation, such as gene expression, mRNA stability, protein folding, cell signaling transduction, cytoskeleton assembly, protein location, protein stability, and protein–protein interaction, even extending to a broad range of cancers through irregular expression of HDACs^9–13^. Microtubules (MTs), a plentiful component of cytoskeletons, are involved in cell division, contribute to intracellular transport for organelle positioning, and regulate cell shape and motility^14^. Acetylation of N-terminal Lsy40 on α-tubulin occurs on the microtubule polymer and is associated with stable microtubule structures and cell morphology^15–17^.

Accumulated evidence has shown that balance in α-tubulin acetylation is primarily regulated by two deacetylases (histone deacetylase 6 [HDAC6] and sirtuin2 [SIRT2])^18–20^ and several acetylases (MEC-17, ELP 3, and Gcn 5)^12,13,21^. Suppression of HDAC6 results in lower microtubule stability and consequently increases cell stress and death. An endogenous HDAC6-knockout cell is resistant to oncogenic transformation, and inactivation of HDAC6 can reduce tumour formation in mice^22^. Moreover, transforming growth factor-β (TGF-β)-induced EMT is associated with HDAC6-dependent deacetylation of α-tubulin in A549 lung adenocarcinoma cells, and acetylated α-tubulin serves as a novel regulator and marker of EMT^1,23^. However, how tubulin acetylation induces cell death and subsequently suppresses tumour progression remains poorly understood. Tubulin acetylation controls the dynamics of focal adhesion for lamellipodial extension during cell migration. Thus, altered α-tubulin acetylation may be involved in cancer cell migration, invasion, and cancer metastasis.

MEC-17/aTAT, a newly discovered acetyltransferase, is highly conserved from *Tetrahymena* to mammalian species^24,25^. Functionally, MEC-17 can directly promote α-tubulin acetylation *in vitro* and serves as the major α-tubulin acetyltransferase *in vivo*^25^. MEC-17 colocalizes with cortactin at the cell adherent surface and is essential for two-dimensional (2D) migration and invasive migration of MDA-MB-231 cells in a collagen matrix, indicating that MEC-17 is involved in tumour metastasis^26^. However, although MEC-17 is an α-tubulin acetyltransferase for tubulin PTM, its effects on cell physiology as well as the molecular mechanism underlying its role in cancer cell morphology and motility through regulation of EMT and cell polarity remain poorly understood.

In this study, we demonstrated that MEC-17 inhibits cell motility by increasing spreading area and reducing the number of pseudopodial protrusions. Stably expressing MEC-17 cells, which showed asthenic tumour metastatic ability, exhibited suppression of EMT, redistribution of cell polarization, higher cdc42 activity, and downregulation of Rho-GAP, ARHGAP21. Inhibition of cdc42 activity restored MEC-17-induced EMT repression, cell polarization disorientation, and migration attenuation. In addition, silencing endogenous MEC-17 increased the numbers of pseudopods and caused aggressive movement. Despite the crucial role of MEC-17 in α-tubulin acetylation for MTs stability and cytoskeletal organization, MEC-17 could be a novel regulator of EMT process through polarity modification and small GTPase activity.

## Results

### Overexpression of MEC-17 enhances α-tubulin acetylation and cell spreading area but inhibits pseudopod formation in a 3D culture

**Figure 1:**
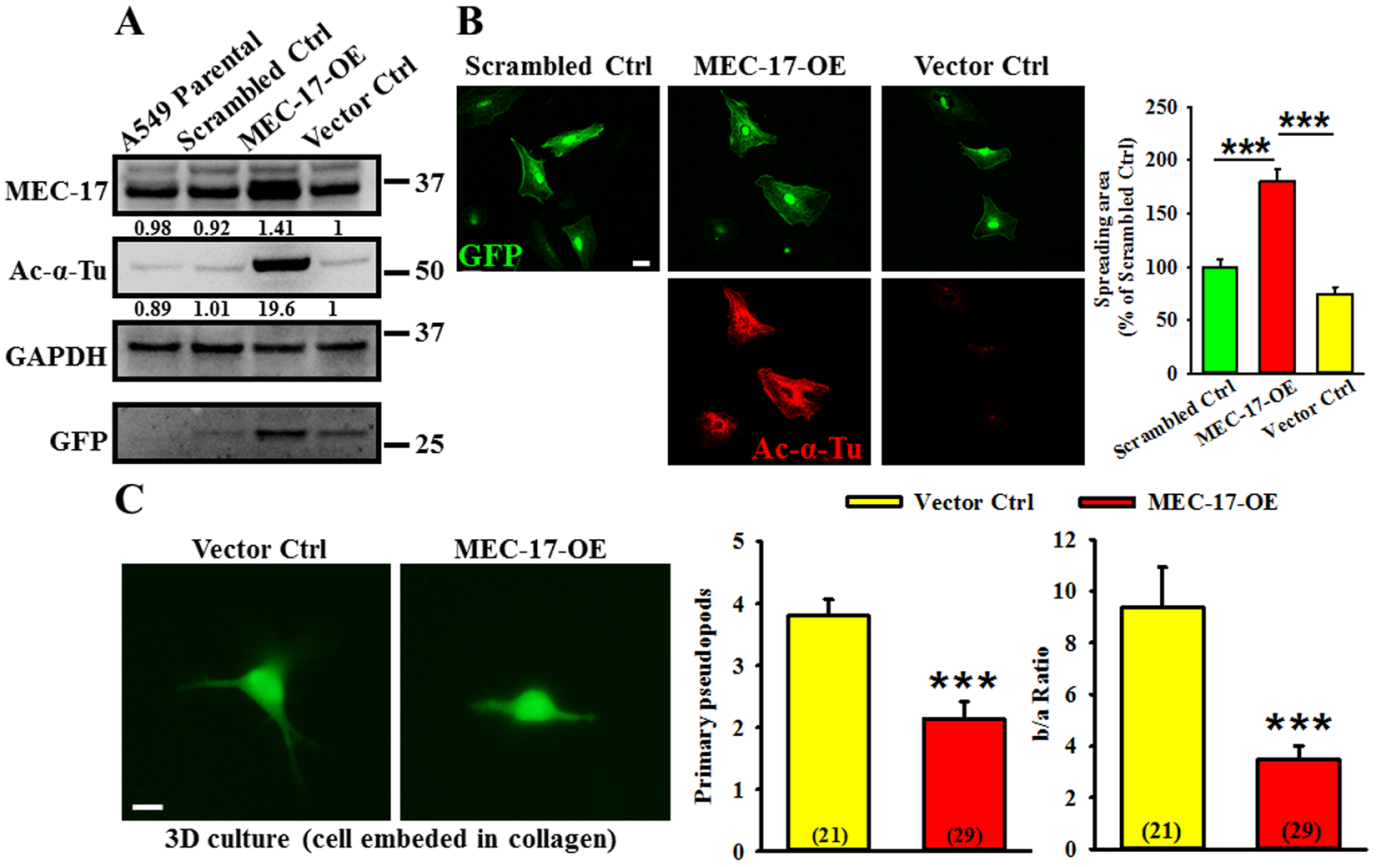
Overexpression of MEC-17 increases α-tubulin acetylation and cell spreading area but decreases pseudopods. (**A**) Western blot analysis shows the protein level of MEC-17 and tubulin acetylation in untransduced (A549 parental) and vector control (vector Ctrl), scrambled control (scrambled Ctrl), and MEC-17-transduced A549 cells (MEC-17-OE). The expression of MEC-17 in the MEC-17-transduced A549 cells is the highest. GAPDH served as a loading control, and GFP was present in the transduced control. The relative protein intensities were shown. Uncropped blots are displayed in the supplementary information (Supplementary Fig. S2). (**B**) The representative immunofluorescent images show the cell morphology and level of tubulin acetylation. A549 cells were transduced with vector control, scrambled control, or MEC-17-overexpressed plasmid DNA encoding with GFP. The Ac-α-Tu signals were presented as MEC-17 overexpressed cells. Scale bar, 10 μm. The representative bar graph showing the quantification of the cell spreading area demonstrates that MEC-17 overexpression increased the cell spreading area compared with the scrambled and vector control groups. ***P < 0.001 in a one-way ANOVA. (**C**) The representative GFP immunofluorescent images show the morphology of A549 cells embedded in collagen (3D culture) transduced with the MEC-17 construct or a vector control. Scale bar, 10 μm. The representative bar graphs showing the quantification of primary pseudopods and pseudopod extension (b/a ratio) demonstrate that MEC-17 overexpression reduced the pseudopod number and extension compared with the vector control group. (n = 21 for vector control; n = 29 for MEC-17-overexpression). ***P < 0.001 in a one-way ANOVA.

To understand the detailed function of MEC-17 in cancer cells, we determined its role in the A549 cell line through gene overexpression or knockdown methods. Western blot analyses of lysates of A549 cells stably expressing scrambled control, vector control, or MEC-17-overexpression constructs were performed. As shown in Fig. 1A, significantly increased protein expression in the MEC-17-overexpressed group was noted compared with that in the parental, scrambled control, and vector control groups. In addition, the level of tubulin acetylation was higher in MEC-17-overexpressed cells in both Western blotting (Fig. 1A) and immunofluorescent examinations (Fig. 1B). Because an increase in α-tubulin acetylation can increase the cell adhesion area^27^, we examined whether MEC-17 overexpression affects cell morphology. Our results revealed that the spreading area as measured using the GFP signal profile in the MEC-17-overexpressed A549 cells was larger than that in the scrambled control and vector control groups (Fig. 1B). Besides, 3D matrices cultivation is a superior system for representing mesenchymal mode characterized by an elongated shape with protruding pseudopods^28^. Thus, we further used the 3D culture system by embedding cells into type I collagen to investigate whether the cell morphology had been altered by MEC-17 overexpression. As shown in Fig. 1C, the embedded cells in collagen were grown with pseudopod structures and the number of primary pseudopods and the extension area of pseudopods (b/a ratio, square area surrounding the cell body with (b) or without (a) pseudopods) were smaller in the MEC-17-overexpressed group than in the vector control group. These data indicated that MEC-17 played a critical role in the regulation of cell morphology.

### Overexpression of MEC-17 attenuates cell migration, invasion, and tumour metastasis

**Figure 2:**
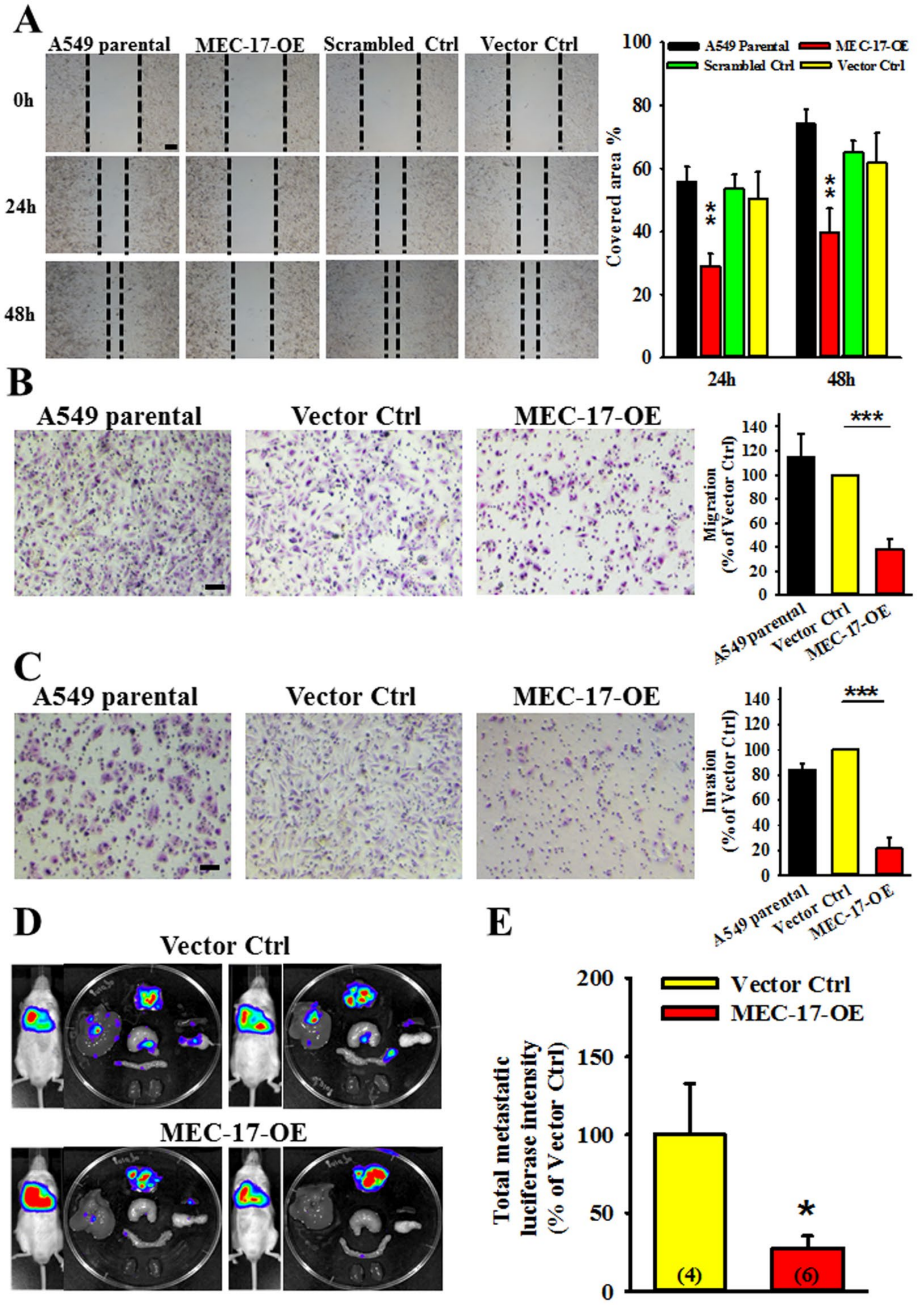
Overexpression of MEC-17 attenuates cell migration and invasion but also tumour metastasis. (**A**) The representative images of wound-healing assay showing the cell-covered area of A549 cells untransduced or transduced with vector control, scrambled control, or MEC-17-overexpressed constructs after scratching with a pipette tip for 0, 24, and 48 h revealed that MEC-17 attenuates cell migration ability. The representative bar graphs showing the quantification of cell migration ability were displayed by measuring the distance between the front edge of cell movement for each cell condition at 24 and 48 h. The percentage of the covered area represents the degree of cell migration. Overexpression of MEC-17 downregulated cell migration ability compared with parental, scrambled, and vector control cells. Scale bar, 100 μm. **P < 0.01 in a one-way ANOVA. (**B**) The representative images of Transwell assay showing the A549 cell untransduced or transduced with vector control or MEC-17-overexpressed constructs penetrated the lower surface of the filter stained with Giemsa. MEC-17-overexpressed A549 cells exhibited inhibition of migration. Scale bar, 100 μm. The representative bar graph showing the quantification of migratory cells demonstrated that overexpression of MEC-17 decreased cell migration compared with the vector control group. ***P < 0.001 in a one-way ANOVA. (**C**) The representative images of Transwell assay showing the A549 cell untransduced or transduced with vector control and MEC-17-overexpressed constructs penetrated through the Matrigel to the other surface of the filter and stained with Giemsa. MEC-17-overexpressed A549 cells show suppression of invasion. Scale bar, 100 μm. The representative bar graph showing the quantification of invasive cells demonstrated that overexpression of MEC-17 decreased cell invasion compared with the vector control group. ***P < 0.001 by one-way ANOVA. (**D**) The representative bioluminescent images of tumour growth and metastasis in orthotopic lung cancer model for 4 weeks of inoculation show the A549-Luc C8 cell stably expressing vector control (upper panels) or MEC-17-overexpression (bottom panels). (**E**) The representative bar graphs showing the quantification of tumour metastatic ability were displayed by measuring the luciferin signals. MEC-17 attenuated tumour metastatic ability compared with the vector control group. *P < 0.05 in a one-way ANOVA.

Because MEC-17 regulated cell morphology, we investigated whether the increase in the cell spreading area or decrease in the pseudopod number influenced cell migration. As shown in Fig. 2A, the wound-healing assay showed that cell motility was lower in MEC-17-overexpressed cells compared with parental cells and the scrambled control or vector control groups. To examine whether the MEC-17-induced lower cell motility was due to inhibition of cell proliferation, we evaluated the natural cell growth assay of A549 parental, vector control and MEC-17-overexpressed cells to determine the doubling time (DT). The calculated DT of A549 parental, vector control and MEC-17-overexpressed cell was 24.15 ± 0.57, 23.15 ± 0 and 25.8 ± 1.63 hrs, respectively. Therefore, overexpression of MEC-17 was not affected cell proliferation. Furthermore, MEC-17-attenuated cell migration was confirmed using the Transwell migration assay. Fewer cells penetrated the membrane filter in MEC-17-overexpressed A549 cells than in the parental and vector control groups (Fig. 2B). Subsequently, we examined the invasive ability of MEC-17-overexpressed A549 cells by using the Matrigel-coated Transwell assay. As shown in Fig. 2C, the invasive ability of MEC-17-overexpressed cells was lower than that of the parental or vector control cells. Similar results were obtained in stable MEC-17-overexpressed MDA-MB-231 cells compared with parental and vector control cells (Supplementary Fig. S1). We further determined whether the MEC-17-induced suppression of cell movement and pseudopod formation could suppress tumour metastasis *in vivo*. An orthotopic animal model generated by inoculating luciferase-expressing A549 cells into the right lung was evaluated by the IVIS system at 4 weeks. Our results revealed that the intensity of signaling in organs, including the liver, stomach, duodenum, kidney, pancreas, and spleen, was lower in the MEC-17-overexpressed group than in the vector control group (Fig. 2D and E). These results collectively demonstrate the inhibitory effect of MEC-17 on cell migration and invasion.

### Loss of endogenous MEC-17 can induce pseudopod formation and promote cell migration and invasion

**Figure 3:**
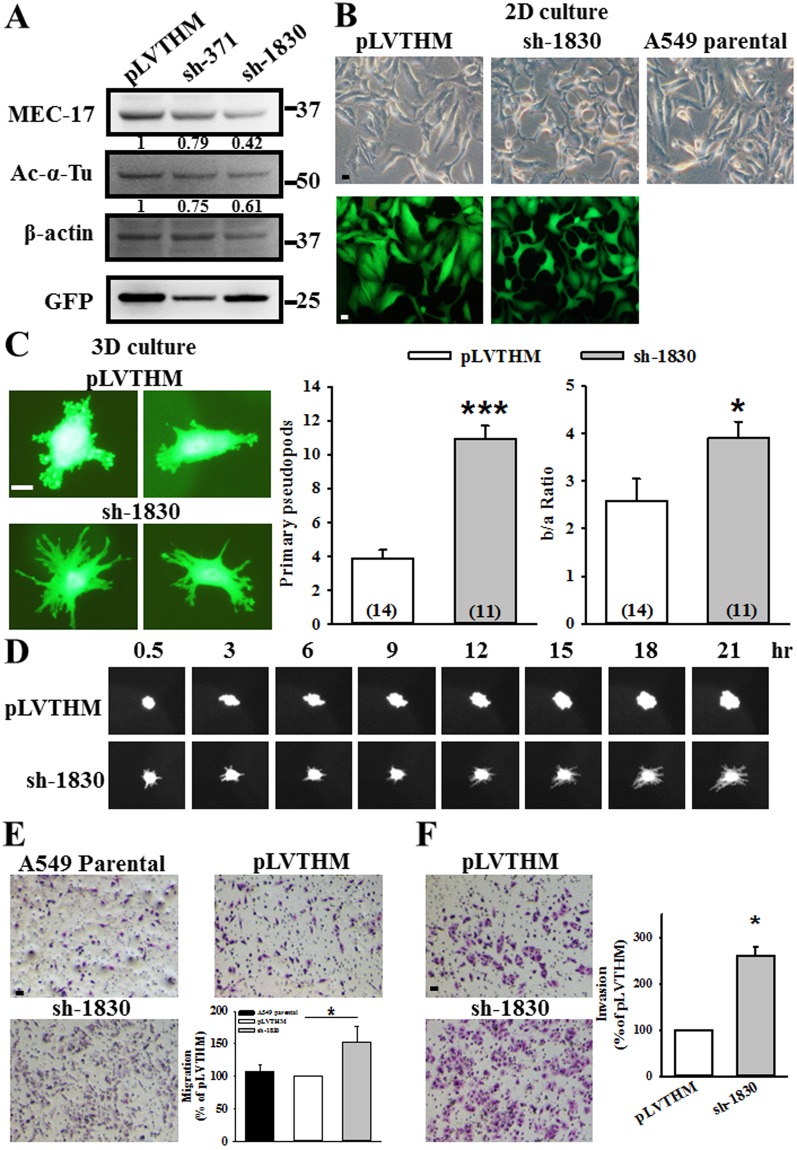
Loss of endogenous MEC-17 induces pseudopods formation and promotes cell migration and invasion. (**A**) Western blot analysis showed the protein level of MEC-17 and tubulin acetylation, in vector control (pLVTHM) and two MEC-17 shRNA transduced cells (sh-371, sh-1830). Beta-actin served as the internal control. GFP was used as the transduction control. The relative protein intensities were shown. Uncropped blots are displayed in the supplementary information (Supplementary Fig. S2). (**B**) The representative images show the cell morphology of A549 cells transduced with or without pLVTHM or MEC-17-shRNA (sh-1830) plasmid DNA encoding with GFP under the 2D culture condition (top panels, bright field; bottom panels, GFP fluorescence). Scale bar, 10 μm. (**C**) The representative GFP immunofluorescent images showing the morphology of A549 cells stably expressing with the pLVTHM or MEC-17 shRNA (sh-1830) were embedded in collagen (3D). A decrease in MEC-17 induced the elongated morphology with pseudopod protrusions of A549 cells at 24 h. Scale bar, 10 μm. The representative quantified bar graphs of primary pseudopods and pseudopod extension (b/a ratio) show that MEC-17 knockdown enhanced the pseudopod number and extension area compared with the pLVTHM group. (n = 14 for pLVTHM, n = 11 for sh-1830). ***P < 0.001, *P < 0.05 in a one-way ANOVA. (**D**) Time-lapse micrographs showing the dynamic extension and retraction of pseudopod protrusions in the 3D culture. Decrease of MEC-17 increased pseudopod protrusions within 24 h. (**E**) The representative images of Transwell assay showing the A549 cell untransduced or transduced with pLVTHM or sh-1830 constructs penetrated to the lower surface of the filter and stained with Giemsa. Scale bar, 100 μm. The representative bar graph showing the quantification of migratory cells demonstrated that MEC-17 knockdown promoted the migration ability compared with the pLVTHM vector control group. *P < 0.05 in a one-way ANOVA. (**F**) The representative images of the Matrigel-coated Transwell assay show the A549 cell transduced with pLVTHM or sh-1830 constructs penetrated to the lower surface of the filter and stained with Giemsa. Scale bar, 100 μm. The representative bar graph showing the quantification of invasive cells demonstrated that MEC-17 knockdown promoted the invasive ability compared with the pLVTHM vector control group. *P < 0.05 by one-way ANOVA.

The importance of endogenous MEC-17 in the regulation of cell morphology and motility was further determined by transducing A549 cells with lentiviral constructs encoding shRNA against MEC-17. Western blotting analysis revealed significant reductions in MEC-17 expression and acetyl-α-tubulin in A549 cells transduced with MEC-17 shRNA-1830 (sh-1830), as well as slight reductions with shRNA-371 (sh-371) compared with the control pLVTHM vector (Fig. 3A), thereby confirming the effectiveness of shRNA in knocking down MEC-17. Then, we chose sh-1830 for subsequent experiments. We first investigated the effect of decreased endogenous MEC-17 on cell morphology. As shown in Fig. 3B, the MEC-17-knockdown A549 cells exhibited more protrusions in the 2D culture system. In the 3D culture system, the embedded cells grew multiple primary pseudopod protrusions and had a larger extension area in the MEC-17-knockdown group than in the pLVTHM vector control group (Fig. 3C). We then examined the dynamic of pseudopod protrusions by using a time-lapse microscope to monitor A549 cell growth. As shown in Fig. 3D, the pseudopod protrusions quickly extended or retracted and a greater number of protrusions, particularly elongated protrusions, were observed in MEC-17-knockdown cells than in the vector control during cell growth periods (0.5–21 h). The involvement of pseudopod protrusions in the mesenchymal movement hinted that MEC-17-knockdown cells might exhibit aggressive motility. To this end, we further evaluated the migrated and invasive ability of MEC-17-knockdown A549 cells through Transwell analysis. Our results showed that the migration and invasive ability of MEC-17-knockdown cells were higher than those of the pLVTHM vector and parental cells (Fig. 3E and F).

### MEC-17 induces EMT inhibition

**Figure 4:**
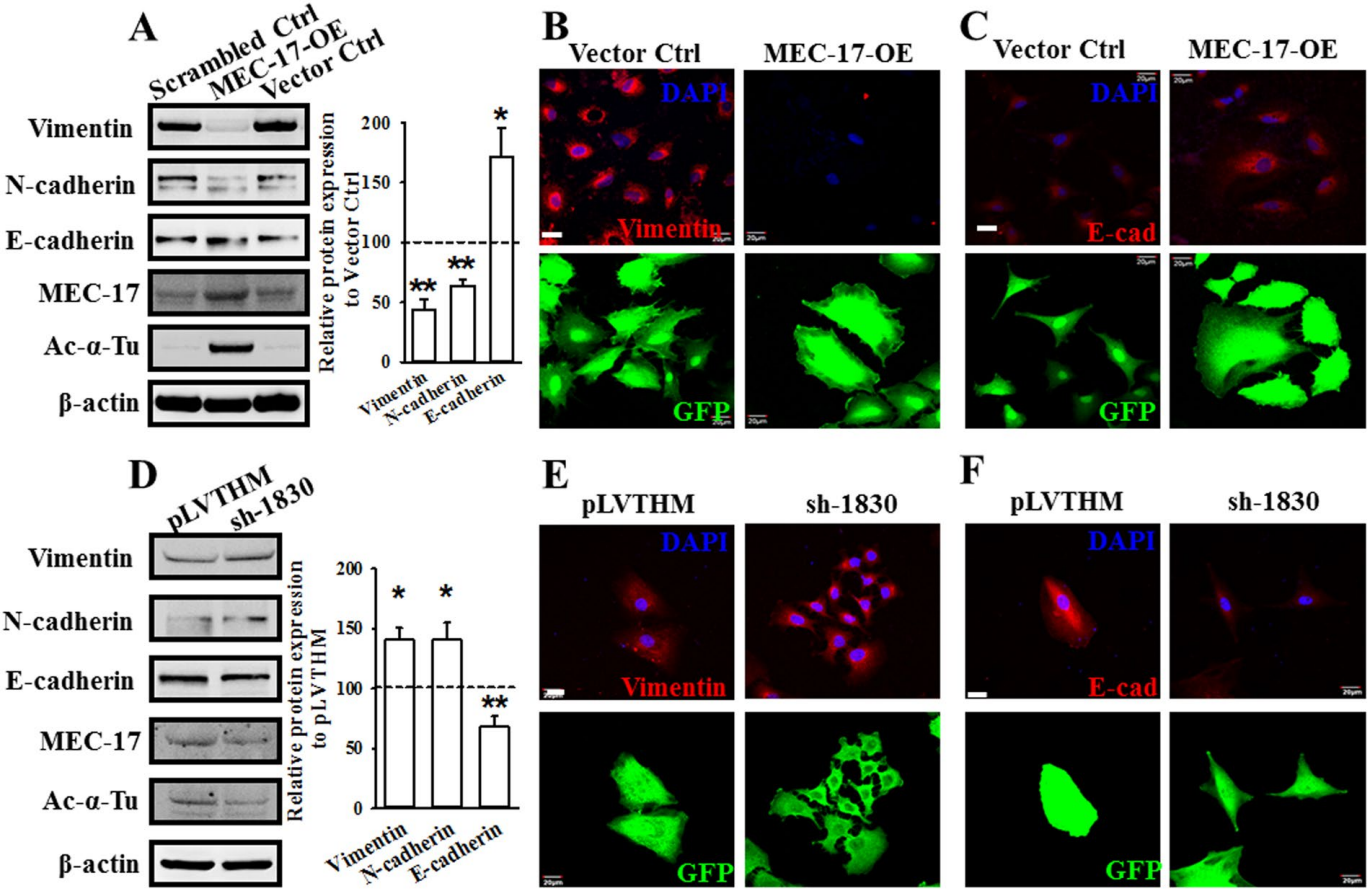
MEC-17 modulates cell motility through EMT suppression. (**A**) Western blots showed the protein levels of vimentin, N-cadherin, E-cadherin, MEC-17, and acetyl-α-tubulin in scrambled control or vector control and MEC-17-overexpressed A549 cells. Beta-actin served as the internal control. Representative bar graph quantification depicting levels of vimentin, N-cadherin and E-cadherin showed the repression of EMT process in MEC-17-overexpressed A549 cells. *P < 0.05, **P < 0.01 in a one-way ANOVA. Uncropped blots are displayed in the supplementary information (Supplementary Fig. S3). (**B**) The representative doubling immunofluorescence images show the intensity of vimentin (red, top panels) and cell morphological marker GFP (bottom panels) in A549 cells stably expressing the vector control or MEC-17-overexpressed constructs. Scale bar, 20 μm. (**C**) The representative doubling immunofluorescence images show the intensity of E-cadherin (E-cad, red, top panels) and cell morphological marker GFP (bottom panels) in A549 cells stably expressing the vector control or MEC-17-overexpressed constructs. Scale bar, 20 μm. (**D**) Western blots show the protein levels of vimentin, N-cadherin, E-cadherin, MEC-17, and acetyl-α-tubulin from pLVTHM and MEC-17-knockdown (sh-1830) A549 cells lysts. Beta-actin served as the internal control. Representative bar graph quantification depicting amounts of vimentin, N-cadherin and E-cadherin showed the promotion of EMT process in MEC-17-knockdown A549 cells. *P < 0.05, **P < 0.01 in a one-way ANOVA. Uncropped blots are displayed in the supplementary information (Supplementary Fig. S3). (**E**) The representative doubling immunofluorescence images show the intensity of vimentin (red, top panels) in A549 cells stably expressing the pLVTHM or sh-1830 constructs. The GFP fluorescence images (bottom panels) were used for transduction control and morphology of A549 cells. Scale bar, 20 μm. (**F**) The representative doubling immunofluorescence images show the intensity of E-cadherin (E-cad, red, top panels) in A549 cells stably expressing the pLVTHM or sh-1830 constructs. The GFP fluorescence images (bottom panels) were used for transduction control and morphology of A549 cells. Scale bar, 20 μm.

TGF-β was reported to induce EMT through HDAC6-dependent deacetylation of α-tubulin in lung adenocarcinoma A549 cells; thus, α-tubulin acetylation could serve as a marker for EMT^1^. We determined whether MEC-17 was involved in the regulation of EMT. As shown in Fig. 4A, the protein levels of vimentin and N-cadherin, markers of the mesenchymal phenotype, decreased dramatically in MEC-17-overexpressed A549 cells compared with the scrambled and vector control cells, whereas the expression level of E-cadherin in MEC-17-overexpressed cells was higher than that in scrambled and vector control cells. Furthermore, the results of the confocal microscopic immunofluorescent analysis were consistent with those of Western blot analysis (Fig. 4B and C). MEC-17 knockdown increased the expression of vimentin and N-cadherin and decreased that of E-cadherin (Fig. 4D–F). These data suggest that MEC-17 plays a critical role in EMT process.

### MEC-17 disrupts cell polarity through redistribution of GM130

**Figure 5:**
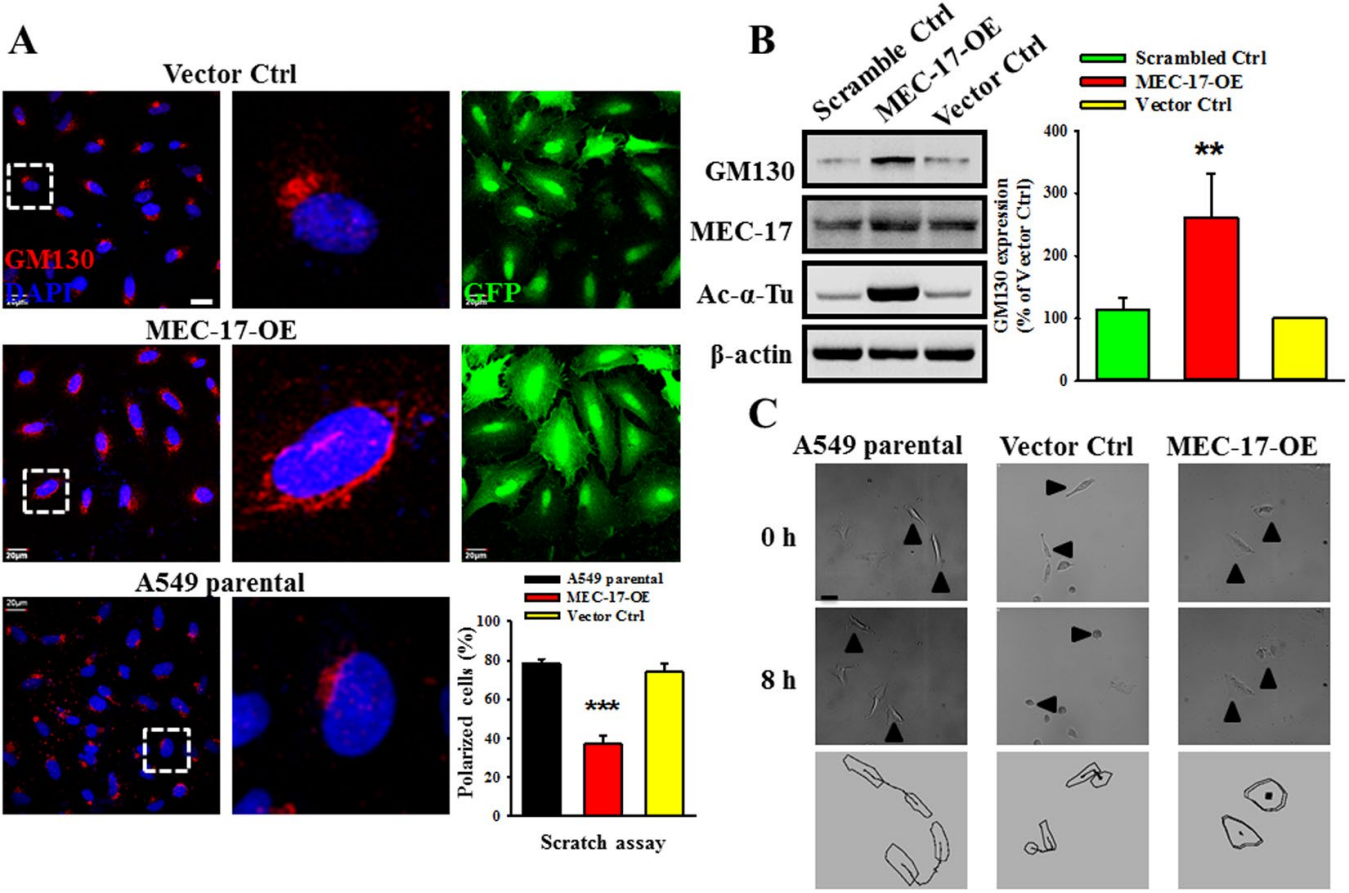
Overexpression of MEC-17 disrupts the specification of cell polarity and increases GM130 expression. (**A**) The representative immunofluorescence images showing the A549 cells untransduced or transduced with vector control, or MEC-17-overexpressed constructs after scratching with a pipette tip for 24 h were immunostained with antibodies to the Golgi marker (GM130), GFP and nucleus marker, DAPI. Red, green, and blue staining corresponds to GM130, GFP, and DAPI staining, respectively. Scale bar, 20 μm. Representative magnified images were displayed the dispersed and randomized in orientation of GM130 protein staining in MEC-17-overexpressed A549 cells compared with the vector or parental groups. The quantitative analysis of GM130-condensed cells along the scratch area was shown in the bar graph plot. The proportion of polarized cells in MEC-17-overexpressed A549 cells was lower than that in the vector and parental control groups. ***P < 0.001 in a one-way ANOVA. (**B**) Western blots show the protein levels of GM130, MEC-17, acetyl-α-tubulin from scrambled control, vector control, and MEC-17-overexpressed A549 cell lysts. MEC-17 and Ac-α-tubulin were used to control MEC-17 overexpression. Beta-actin served as the internal control. The representative bar graph shows the quantified intensity of GM130 expression in A549 cells stably expressing scrambled control, vector control, and MEC-17-overexpressed constructs. **P < 0.01 in a one-way ANOVA. Uncropped blots are displayed in the supplementary information (Supplementary Fig. S3). (**C**) The time-lapse microscopic images showing the dynamic cell motility and corresponding trajectory illustrate that MEC-17 overexpression inhibited cell movement compared with the parental and vector control groups within 8 h. Scale bar, 20 μm.

Previous study showed that epithelial cells undergoing EMT exhibited loss of cell–cell adhesion and polarity alteration^29^. The Golgi apparatus is linked to cell polarity, and GM130, a Golgi matrix protein, is a regulator of cell polarity. Thus, we examined whether MEC-17 overexpression affected the localization and expression profile of GM130. As shown in Fig. 5A, GM130 was dispersedly and randomly distributed around the cell nucleus in MEC-17-overexpressed A549 cells, in contrast to vector control and parental cells. The amount of polarized cells reduced significantly in MEC-17-overexpressed cells compared with parental and vector control cells. Moreover, we found that the protein level of GM130 increased in MEC-17-overexpressed cells compared with scrambled and vector control cells (Fig. 5B). To verify whether MEC-17-disturbed cell polarity affected cell migration ability, we monitored single cell motility with a time-lapse microscope. As shown in Fig. 5C, the MEC-17-overexpressed cells exhibited stiffness and a lower moving trajectory compared with the parental and vector control cells. These data demonstrated that MEC-17 overexpression affects the cell polarity required for directional cell migration.

### MEC-17 influences cell polarity through enhancement of cdc42 activity

**Figure 6:**
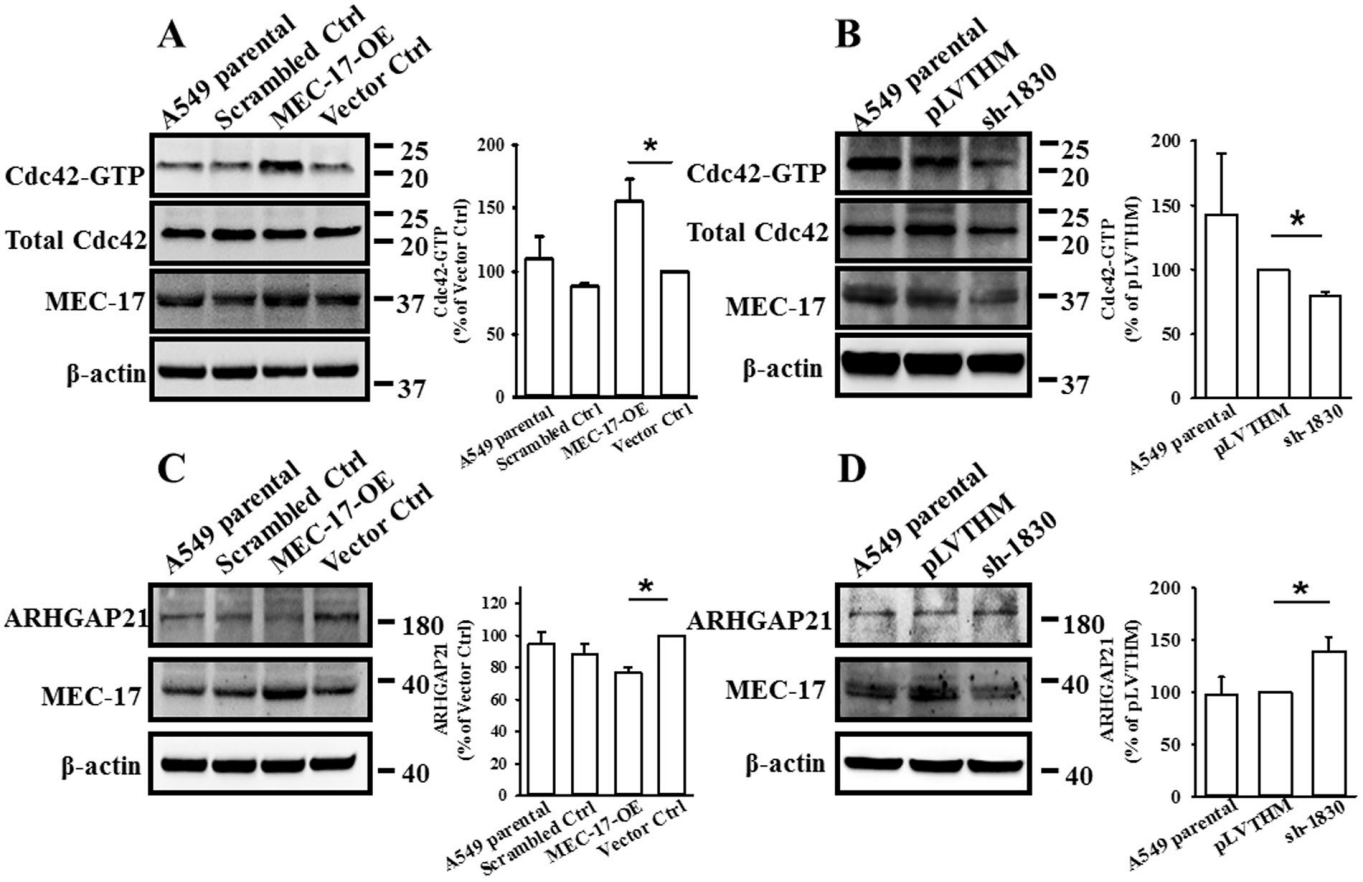
MEC-17 can increase cdc42 activity and suppress ARHGAP21 expression. (**A**) Representative Western blots and corresponding bar graph quantification show the level of cdc42-GTP (cdc42 activity) in parental, scrambled control, vector control, and MEC-17-overexpressed A549 cells. The total cdc42 and MEC-17 were used as internal and transduced controls, respectively. Beta-actin served as the loading control to display equal amounts of protein. *P < 0.05 in a one-way ANOVA. Uncropped blots are displayed in the supplementary information (Supplementary Fig. S4). (**B**) Representative Western blots and corresponding bar graph quantification show the level of cdc42-GTP (cdc42 activity) in parental A549 cells or stably expressing pLVTHM or MEC-17-shRNA groups. *P < 0.05 in a one-way ANOVA. Uncropped blots are displayed in the supplementary information (Supplementary Fig. S4). (**C**) The representative Western blots and corresponding bar graph quantification show the expression of ARHGAP21 in parental, scrambled control, vector control, and MEC-17-overexpressed A549 cells. Beta-actin served as the internal control. *P < 0.05 in a one-way ANOVA. Uncropped blots are displayed in the supplementary information (Supplementary Fig. S4). (**D**) Representative Western blots and corresponding bar graph quantification show the expression of ARHGAP21 in parental A549 cells or stably expressing pLVTHM and MEC-17-shRNA groups. *P < 0.05 in a one-way ANOVA. Uncropped blots are displayed in the supplementary information (Supplementary Fig. S4).

Endogenous cdc42 was reported to act as a negative regulatory function on intrinsic migration or invasion of some aggressive breast cancer cells^30^. We then examined the expression and activity of cdc42; the results revealed that the amount of active GTP-bound cdc42 (cdc42-GTP), an indicator of cdc42 activity, was significantly increased in MEC-17-overexpressed cells compared with scrambled control, vector control, and parental cells (Fig. 6A). By contrast, loss of MEC-17 reduced the level of cdc42-GTP in MEC-17-knockdown A549 cells compared with the parental and pLVTHM vector control groups (Fig. 6B). ARHGAP21, a Cdc42-specific GTPase-activating protein, was involved in cell migration, α-tubulin acetylation in EMT, and microtubule- and dynein-dependent Golgi motility and positioning^31,32^. Therefore, we hypothesized that cdc42 activity is regulated by ARHGAP21 after manipulation of MEC-17. We found that the level of ARHGAP21 was reduced in MEC-17-overexpressed cells compared with the scrambled control, vector control, and parental groups, whereas MEC-17 knockdown increased its expression compared with the parental and pLVTHM vector control groups (Fig. 6C and D). These results demonstrated that MEC-17-induced inhibition of migration was associated with the dysregulation of Golgi orientation and cdc42-dependent actin dynamics disturbance.

### Inhibition of cdc42 activity rescues EMT suppression, enhancement of cell spreading area, and cell migration attenuation in MEC-17-overexpressed cells

**Figure 7:**
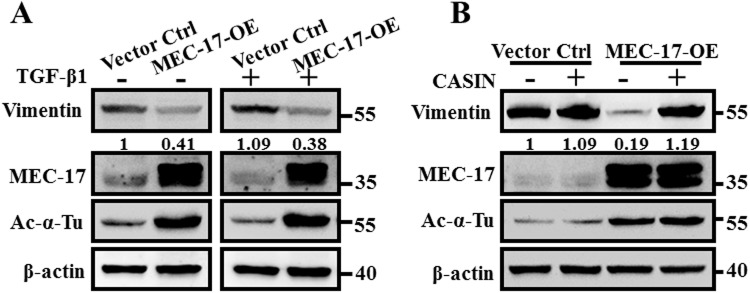
Pharmacological inhibition of cdc42 activity rescued MEC-17-induced suppression of EMT. (**A**) The representative Western blots show the protein levels of vimentin, MEC-17, and acetyl-α-tubulin in vector control and MEC-17-overexpressed A549 cells after treatment with TGFβ1 (20 ng/mL) for 48 h. Beta-actin served as the internal control. The relative vimentin intensities normalized to vector control group were shown. Uncropped blots are displayed in the supplementary information (Supplementary Fig. S5). (**B**) The representative Western blots show the protein levels of vimentin, MEC-17, and acetyl-α-tubulin in vector control or MEC-17-overexpressed A549 cells after treatment with the cdc42 inhibitor CASIN (5 μM) for 48 h. Beta-actin served as the internal control. The relative vimentin intensities normalized to vector control group were shown. Uncropped blots are displayed in the supplementary information (Supplementary Fig. S5).

**Figure 8:**
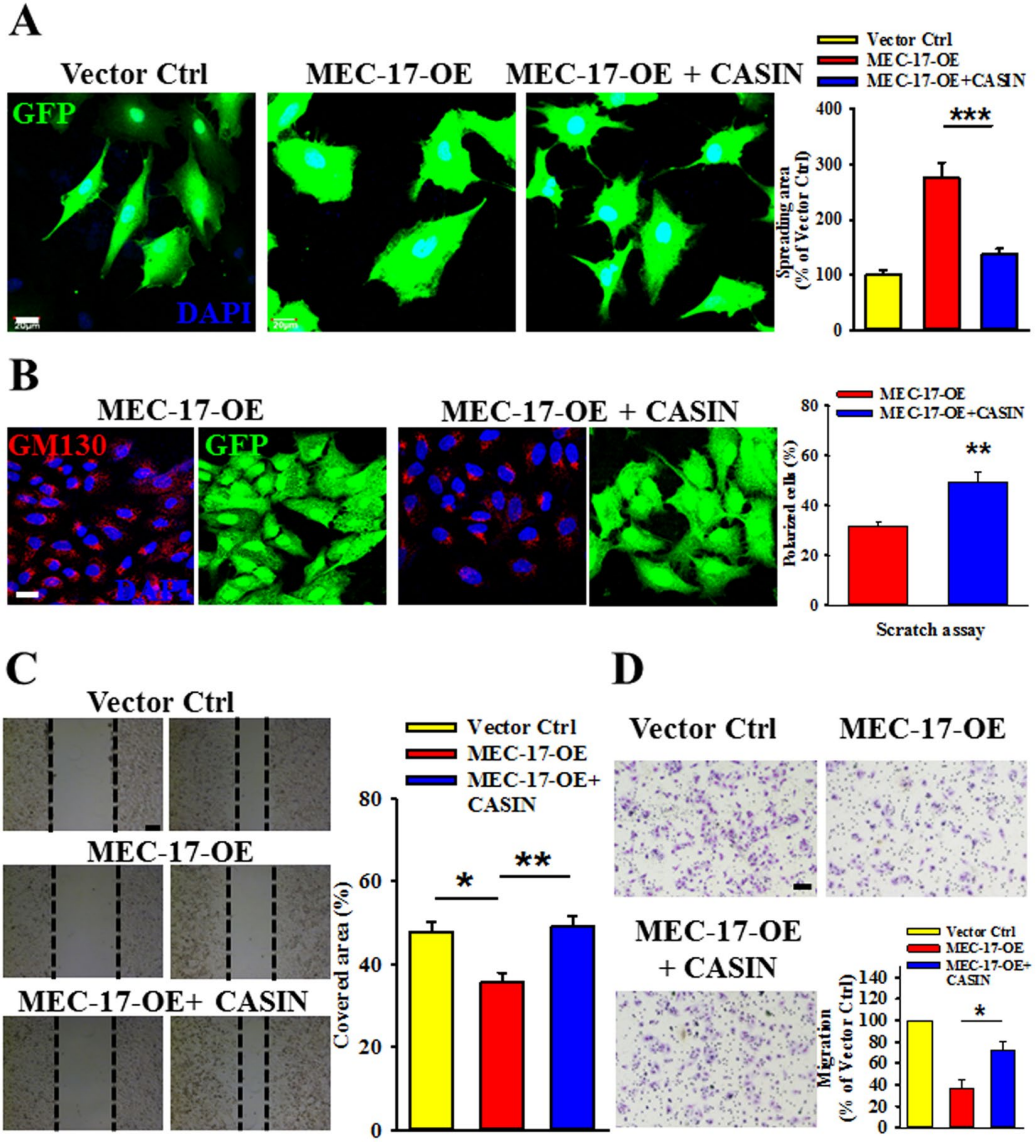
Blockage of cdc42 activity restored MEC-17-induced cell polarity disruption, spreading area enhancement, and migration attenuation. (**A**) The representative immunofluorescent images show the cell morphology of A549 cells transduced with vector control or MEC-17-overexpressed plasmid DNA in the presence or absence of CASIN. Scale bar, 20 μm. The representative bar graph showing the quantification of the cell spreading area demonstrated that inhibition of cdc42 activity abolished the MEC-17-induced increased cell spreading area compared with the MEC-17-overexpressed group. ***P < 0.001 in a one-way ANOVA. (**B**) The representative immunofluorescence images showing the A549 cells transduced with MEC-17-overexpressed constructs in the presence or absence of CASIN after scratching with a pipette tip for 24 h were immunostained with antibodies of GM130, GFP, and DAPI. Scale bar, 20 μm. The representative bar graphs showing the quantitative analysis of GM130-condensed cells along the scratch area illustrated that the lower proportion of polarized cells in MEC-17-overexpressed A549 cells is rescued under application of the cdc42 inhibitor CASIN. **P < 0.01 in a one-way ANOVA. (**C**) The representative images of wound-healing assay showing the cell-covered area of A549 cells transduced with vector control or MEC-17-overexpressed constructs in the presence or absence of CASIN after scratching with a pipette tip at 0 and 48 h demonstrated that diminished cdc42 activity blocked the MEC-17-induced attenuation of cell migration. The representative bar graphs showing the quantification of cell migration ability were displayed by measuring the distance between the front edge of cell movement for each cell condition at 48 h. Scale bar, 100 μm. *P < 0.05, **P < 0.01 in a one-way ANOVA. (**D**) The representative images of Transwell migration assay showing the A549 cell transduced with vector control or MEC-17-overexpressed constructs in the presence or absence of CASIN penetrated the lower surface of the filter stained with Giemsa. Scale bar, 100 μm. The representative bar graph showing the quantification of migratory cells demonstrated that suppression of cdc42 activity restored MEC-17-induced inhibition of cell migration. ***P < 0.001 in a one-way ANOVA.

The TGF-β family signaling pathway is essential for EMT induction and is accompanied by HDAC6-dependent deacetylation of α-tubulin, leading to decreased MT stability, and TGF-β stimulates EMT by disrupting basoapical polarity in polarized epithelial cells^1,23,33–35^. Therefore, we investigated whether the MEC-17-induced EMT suppression could be rescued by treatment of TGF-β1. Against our expectations, the level of vimentin was not substantially recovered compared with the vector control group (Fig. 7A), suggesting that MEC-17 overexpression may occlude TGF-β1-induced EMT-associated gene expression and that cdc42 activity disturbance may be the major underlying mechanism downstream of MEC-17. CASIN, a selective cdc42 activity inhibitor, has been reported to reduce the elevated level of active cdc42^36^. We assessed whether CASIN application could restore the decreased expression of vimentin in MEC-17-overexpressed cells, and found that it could (Fig. 7B); this suggested that cdc42 activity is essential to MEC-17-induced EMT suppression. To this end, we further determined the importance of cdc42 activity in enhancing the MEC-17-induced spreading area, Golgi orientation, and migration ability. Our results demonstrated that MEC-17-induced enlargement of the cell spreading area and dispersal and redistribution of GM130 were blocked after pretreatment of CASIN compared with the MEC-17-overexpressed group (Fig. 8A and B). Moreover, MEC-17-induced attenuation of cell migration was rescued after application of CASIN by the wound healing and Transwell assay (Fig. 8C and D), suggesting that MEC-17 determined cell morphology, migration and invasion ability, and tumour metastasis through ARHGAP21-mediated cdc42 activation.

## Discussion

Most cancers are consequences of dysregulation in cell proliferation, morphogenesis, and migration. These cellular malfunctions have been associated with microtubule structural stability^22^. Acetylation of Lsy40 on α-tubulin was the second tubulin PTM to be discovered in early 1985^11^, and recent studies have shown that deacetylase (HDAC6/SIRT2) and acetylase (MEC-17/ATAT1) are considered regulators for controlling the acetylation of α-tubulin^24–26^. HDAC6 is reportedly associated with tumorigenesis and cancer cell survival, and HDAC6 can deacetylate α-tubulin in TGF-β-induced EMT, thereby decreasing MT stability^1,23^. Therefore, downregulation of α-tubulin acetylation is involved in cancer cell growth and tumour progression. To this end, promotion of acetylation of α-tubulin by acetylases may affect the physiological regulation of morphological cell growth and motility. MEC-17, which has been identified as an α-tubulin acetyltransferase, is associated with instability of microtubule-rich structures such as cilia and axons, dentate gyrus distortion, and cortical neuron migration^37–39^. Boggs *et al*. had previously mentioned that elevated levels of α-tubulin acetylation in suspended tumour cells culture condition are a sufficient cause of metastatic potential^40^. Nevertheless, physiological research into MEC-17 and the molecular mechanism underlying the morphological and migration regulation of MEC-17-induced microtubule acetylation remain elusive.

In this study, we verified that MEC-17 inhibits cancer cell motility by increasing the spreading area for adhesion ability and reducing pseudopodial protrusions. In addition, overexpression of MEC-17 inhibited tumour metastasis, suppressed EMT, and disturbed cell polarization through cdc42 activation resulting from decreased expression of Rho-GAP, ARHGAP21. Blockage of cdc42 activity could reverse all of these changes. Notably, loss of endogenous MEC-17 led to a marked increase in pseudopodial protrusions and promoted cell movement. These results highlight the physiological importance of MEC-17 in cancer cell growth and movement correlated with EMT, cell polarity, and small GTPase activity.

Dynamic actin-dependent pseudopodial protrusions are critical for mesenchymal (tumour) cell migration and invasion, and thus also critical for cancer metastasis^41–43^. Our results revealed that MEC-17 overexpression reduced incidence of pseudopodial protrusions and attenuated tumour metastasis *in vivo*, whereas MEC-17 knockdown enhanced pseudopod formation; this suggests that MEC-17 serves as a structural modulator for actin polymerization and depolymerization. Studies have demonstrated that the expression of regulators of actin reorganization is essential for pseudopod formation and associated with tumour cell migration and invasion, such as Wiskott–Aldrich syndrome protein (WASP) family proteins, the Arp2/3 complex, Eps 8, α-actinin, fascin, filamin, LIM kinase/cofilin, and cortactin^42,44,45^. In addition, tumour cell pseudopod-specific proteins (AHNAK, Septin-9, eIF4E, and S100A11) were identified from the transcriptome/proteome analysis and closely for pseudopod formation, actin cytoskeleton dynamics, and tumour cell migration and invasion^41^. One possible means through which this effect is achieved is the influence on pseudopod formation through directly acetylated pseudopod-specific proteins or an indirectly acetylated transcription factor or repressor to promote or hinder pseudopod-specific protein production. Although we did not identify which specific proteins are acetylated by MEC-17, we believe that MEC-17 overexpression can drive or repress the expression of genes such as GM130 (Fig. 5B) and ARHGAP21 (Fig. 6C). In our unpublished data, we also found that Erk1/2 phosphorylation was reduced in MEC-17-overexpressed cells, indicating that the alteration of EMT and polarization protein expression may be regulated by the inhibition of MAPK/ERK signaling pathway. Further studies are required to address this hypothesis.

The prerequisite for cell motility is the pseudopodial protrusions formation and retraction of cell membranes driven by reorganization of the cytoskeleton^46,47^. Many studies have investigated the molecular mechanisms involved in cell migration and have demonstrated that Rho small GTPases are key regulators of actin polymerization and dynamic change. The small GTPase proteins, Rac, Rho, and Cdc42, also played roles in the initial signals, leading to the polarization of migration^29,47^ and microtubule-dependent Golgi positioning^32^. Cell migration direction requires polarized alignment of the cytoskeleton; furthermore, the microtubule-organizing center (MTOC) and Golgi are fundamentally positioned facing forward at the leading edge of the cell^32^. The Golgi apparatus has been linked to polarity; studies have illustrated that GM130 is a regulator of cdc42 signaling that regulates cell polarity and provides a potential link between Golgi and cancer initiation^48^. In our study, immunofluorescence revealed GM130 distribution to be disturbed and its level to be increased in MEC-17-overexpressed cells (Fig. 5A), suggesting that high expression of GM130 protein may not limit the specific Golgi position for retaining the apical–basal polarity, eventually resulting in orientation disruption and weak migration ability. In addition, a Golgi-localized GAP for cdc42 regulated the Arp2/3 complex and F-actin dynamics by controlling cdc42 activity^49^, and endogenous cdc42 was able to inhibit intrinsic migration or invasion and alter PKCd, Erk1/2, and PKA phosphorylation in some aggressive breast cancer cells^30^. Our finding that MEC-17 can activate cdc42 followed by a reduction in ARHGAP21 to regulate cell morphology and movement is consistent with previous studies. However, to investigate whether cdc42 activity is affected by α-tubulin acetylation, we used tubastatin-A, a potent selective inhibitor for HDAC6, to increase the acetylation of α-tubulin, thereby bypassing the involvement of MEC-17. Cdc42 activity was not altered (data not shown), suggesting that α-tubulin acetylation is not necessary for MEC-17-induced cdc42 activation.

In conclusion, our findings demonstrated that MEC-17-induced cdc42 activity through the reduction of GAP regulates EMT process, actin reorganization for morphological regulation, cell polarity maintenance, and cell motility.

## Materials and Methods

### Chemicals and antibodies

Rabbit polyclonal anti-MEC-17 (#ab58742), anti-GM130 (#ab52649), and anti-GFP (#ab6556) antibodies were purchased from Abcam (Cambridge, MA). Mouse monoclonal anti-acetylated tubulin (#T7451) and anti-α-tubulin (#T6199) antibodies were obtained from Sigma-Aldrich (St. Louis, MO). Monoclonal anti-actin (#MAB1501), anti-cdc42 (#07–1466), and a Rac1/cdc42 activation assay kit (#17–441) were purchased from Millipore (Temecula, CA). Anti-E-cadherin (#610182) and anti-N-cadherin (#610920) antibodies were purchased from BD Transduction Laboratories. Anti-GAPDH (#2118) antibodies were purchased from Cell Signaling Technology (Beverly, MA). Antibody against vimentin (#sc-32322), anti-ARHGAP21 (#sc-390145), anti-mouse IgG (#sc-2060), and anti-rabbit IgG (#sc-2054) were obtained from Santa Cruz Biotechnology (Santa Cruz, CA). Alexa-Fluor-488-conjugated anti-rabbit IgG (#A11008) and Alexa-Fluor-568-conjugated anti-mouse IgG (#A11004) were purchased from Molecular Probes (Eugene, OR). T4 DNA ligation kit and restriction enzymes were purchased from New England Biolabs (UK) or Thermo Scientific (Rockford, IL). RIPA buffer solution was purchased from Thermo Scientific. Cell culture reagents were obtained from Life Technologies, Inc. (Rockville, MD).

### Cell culture

A549 human lung adenocarcinoma cells expressing (A549-Luc-C8) or not expressing luciferase were maintained in Kaighn’s modification of Ham’s F12 medium (F-12K medium; Gibco) supplemented with 2 mM glutamine, 100 U/mL penicillin, 100 µg/mL streptomycin, and 10% fetal bovine serum (FBS). MDA-MB-231 (human breast adenocarcinoma) cells were grown in RPMI-1640 medium (Gibco) supplemented with 10% FBS, 2 mM glutamine, 100 U/mL penicillin, and 100 µg/mL streptomycin. Human epithelial kidney (293T) cells were maintained in Dulbecco’s modified Eagle’s medium (DMEM; Gibco) supplemented with 10% FBS (Invitrogen, San Diego, CA) at 37 °C in an atmosphere of 95% air and 5% CO_2_.

### DNA constructs

The A549 human lung adenocarcinoma cell cDNA library was used as the template for generating full-length MEC-17 (NM_024909, amino acids 1–333) cDNA. The MEC-17 cDNA was amplified through a polymerase chain reaction (PCR) and subcloned into the BamHI and XhoI restriction sites of a lentiviral vector (UXIE) with bicistronic expression of transgenes and EGFP under the control of a ubiquitin promoter and separated by an internal ribosomal entry site (IRES). In some MEC-17 overexpression experiments, the DsRed cDNA cloned into UXIE was used as scrambled control group. For knockdown experiments, shRNAs of MEC-17 were subcloned into the pLVTHM vector. Two shRNAs targeted at different regions of the human *mec-17* gene coding sequence (sh-371 and sh-1830) were designed as follows: sh-371, GGATGATCGTGAGGCTCATAA (base pairs 371–391) and sh-1830, GGTAGCTAGGTCCCGATATAC (base pairs 1830–1850), predicted by the BLOCK-iT™ RNAi Designer.

### Lentiviral particle production

Engineered self-inactivating recombinant lentiviral particles were used to overexpress or silence the MEC-17 gene in A549 cells. All viruses were produced by cotransfection of lentiviral DNA with two helper plasmids, vesicular stomatitis virus envelope glycoprotein (VSV-G) and Δ8.9, in HEK293T cells. Media containing recombinant lentiviruses were collected twice after transfection for 48 and 96 h and were concentrated by Lenti-X according to the manufacturer’s instruction to obtain a concentrated stock for stable cell production. After centrifugation, pellets were resuspended in A549 cultured media with titers of 10^8^–10^9^ units/mL. To evaluate the overexpression or knockdown of MEC-17 efficiency, the stable cell lysates were harvested for Western blotting analysis.

### Wound healing assay

The monolayer confluent cells were starved for 12–16 h to suppress cell proliferation, and then scraped with a 1-mL pipette tip across a 6-well plate. After wounding, the culture media containing 10% FBS were replaced. Cells were visualized using the inverted microscope after 24 and 48 h of the migration period. The migrated cells were manually quantified by measuring the cell-covered area with image J software. For quantification of polarized cells in the scratch wounding assay, 1 × 10^5^ cells were cultured on 4-well chamber slides. A scratch area for cell migratory direction was then produced by using a 100-μL pipette tip across the confluent monolayer and incubated in a fresh culture medium containing 10% FBS after overnight starvation. After 24 h, the cells were immunostained for the Golgi apparatus using anti-Golgi Matrix protein, GM130. The cell was determined as polarized when stained Golgi apparatus was compact and located primarily in the cytosolic sector facing the wound or direction of migration.

### Transwell migration and invasion assay

For cell invasion and migration, Transwell inserts coated with or without Matrigel were used, respectively. In brief, 200-μL serum-free F12K media containing 1 × 10^5^ A549 cells stably expressing vector, MEC-17-overexpression, pLVTHM or sh-1830 and serum-free RPMI-1640 media containing 1 × 10^5^ MDA-MB-231 cells stably expressing vector and MEC-17-overexpression were seeded onto the inserts with a pore size of 8.0-μm, respectively. The bottom inserts were then filled with 750 μL of complete cell culture media as a chemoattractant. After 20–24 h, the filter membrane of inserts was fixed and stained with Giemsa. Nonmigratory cells were removed by cotton swabs. The underside images of inserts were captured using a Nikon inverted microscope with ×40 magnification. Cell migration or invasion was quantified by counting the number of cells in five random fields, and the inhibitory percentage was performed in relation to the vector control cells.

### Culturing and monitoring cells embedded in collagen matrices

The three-dimensional (3D) culture was performed as previously described^28^. In brief, PureCor bovine collagen solution (Advance Biomatrix) was applied for collagen matrices. To embed cells in collagen, the MEC-17-overexpressed or knockdown and vector control A549 cells were first cultured on plastic dishes. Then, trypsinizing adherent cells with 0.1% trypsin in EDTA at 37 °C for 5 min and collected in the proportion of 0.5 × 10^6^ cells/mL. Subsequently, 1.7 mL of 3 mg/mL chilled PureCor bovine collagen solution was mixed with 1.3 mL of F-12K medium for A549 cells to form a 1.7 mg/mL collagen solution (3 mL). Then, we centrifuged the cell suspensions and replaced the supernatants with the chilled 1.7 mg/mL mixing collagen solution, and thoroughly mixed the cells with the collagen solution. The cell–collagen mixture was polymerized in a cell incubator at 37 °C for 1 h and then covered with a sufficient amount of the serum-containing medium. To observe the pseudopods, the live cells were cultured in 12-well plates and the GFP-positive cells were examined through immunofluorescent microscopy after 24 h or through the cell culture monitoring system (CCM-1.4/XYZ) from 0 to 24 h.

### Cdc42 activation assays

Cdc42 activity was determined using a Rac1/cdc42 activation assay kit as previously described^30,50^. Briefly, cdc42-GTP from various lysates were pulled down using the GST fusion-protein, corresponding to the p21-binding domain (PBD, residues 67–150) of human PAK-1 bonded to agarose beads (PAK1-PBD agarose conjugate) for 12 h at 4 °C. The beads were washed four times with ice cold 1× MLB lysis buffer, resuspended and boiled in SDS sample buffer, and separated on 4–12% SDS-PAGE gradient gels before being subjected to immunoblot with the anti-cdc42 antibody to detect the presence of cdc42-GTP and total cdc42.

### Immunofluorescence staining

Immunofluorescent staining was modified and performed as previously described^50,51^. In brief, the cells grown on CultureSlides (BD Biosciences) were fixed with 4% paraformaldehyde in PBS for 20 min at room temperature. After washing twice with PBS, the cells were permeabilized and blocked simultaneously in a solution containing 3% bovine serum albumin (BSA) and 0.2% Triton X-100 in PBS for 1 h at room temperature. Subsequently, the indicated primary antibodies, namely anti-GFP (1:1000), anti-E-cadherin (1:1000), anti-Vimentin (1:500), and anti-GM130 (1:1000), were added and incubated overnight at 4 °C. After washing with PBS, bound primary antibodies were visualized through incubation of the cells with appropriate Alexa-Fluor-488-conjugated and Alexa-Fluor-568-conjugated secondary antibodies for 1–2 h at room temperature. 4′,6-Diamidino-2-phenylindole (DAPI) was used as a counterstain to visualize the nuclei. The cells were then rinsed extensively in PBS and mounted on ProLong Anti-fade media (Molecular Probes, Eugene, OR). Confocal fluorescent images were obtained using an FV1000 confocal microscope (Olympus) with a 60× or 63× oil immersion lens, namely NA 1.35 (Uplsapo).

### SDS-PAGE and Western blotting

Cells for analysis were scraped and lysed in a lysis buffer (#C2978; Sigma), RIPA buffer solution, or 1× MLB buffer containing the following protein phosphatase and protease inhibitors: 1 mM Na_3_VO_4_, 0.5 mM phenylmethylsulfonyl fluoride (PMSF), and 1× protease inhibitor cocktail. Samples were sonicated and centrifuged at 14000 rpm at 4 °C for 15 min to remove debris. The supernatant was then assayed for total protein concentration by using a BCA protein assay kit (#23225; Pierce). An equal amount of proteins was resuspended in a 1× SDS sample buffer (2% SDS, 12.5 mM EDTA, 1% β-mercaptoethanol, 20% v/v glycerol, 0.02% bromophenol blue, and 50 mM Tris-Cl, pH 6.8) and boiled for 10 min. Each sample was subsequently separated using electrophoresis in 8% or 10% SDS-PAGE polyacrylamide gel or NuPAGE 4–12% Bis-Tris gradient protein gel. Following transfer onto PVDF or nitrocellulose membranes, the blots were blocked in TBS (150 mM NaCl and 10 mM Tris-Cl, pH 7.4) containing 3% BSA and 0.1% Tween 20 for 1 h. Subsequently, the membranes were blotted for 12–16 h at 4 °C with specific primary antibodies. Each blot was probed with horseradish peroxidase-conjugated secondary antibodies for 1 h, and the chemiluminescent signal was conducted using the Western Lightning Plus ECL immunoblotting detection system (Perkin Elmer). Immunoblots were analyzed by densitometry performed using Image J software.

### Metastatic orthotopic mouse models of lung cancer

A549-Luc C8 Cells (2 × 10^6^ cells in 10-μL PBS) were surgically inoculated into the right lungs of 5–6-week-old male NOD-SKID immunodeficient mice (BioLASCO Taiwan or National Laboratory Animal Center, Taipei, Taiwan) via intrathoracic injection. Mouse body weight was measured weekly and primary tumour outgrowth and metastasis were inspected using IVIS every 2 weeks. Lung and other tissue including heart, liver, stomach, duodenum, kidney, spleen, and pancreas were extracted to determine the total tumour metastatic ability at 4 weeks after inoculation. All experimental procedures were performed according to the National Institutes of Health Guide for the Care and Use of Laboratory Animals and were approved by the Institutional Animal Care and Use Committee of National Health Research Institutes.

### Statistical analysis

All analyzed data for each experiment were normalized relative to controls and presented as mean ± standard error of the mean (s.e.m). Differences between means was evaluated through an analysis of variance (ANOVA) with Bonferroni’s post hoc analysis test for multiple comparisons or an unpaired two-tailed Student’s *t* test. In this study, probability values (*P*) < 0.05 was considered statistically significant. *P* values are denoted with asterisks as follows: *P < 0.05, **P < 0.01, and ***P < 0.001.

## Electronic supplementary material

Supplementary Information
