# Supplementary material for: Alpha-tubulin acetyltransferase/MEC-17 regulates cancer cell migration and invasion through epithelial–mesenchymal transition suppression and cell polarity disruption
Source: Sci Rep. 2018 Nov 30;8:17477. doi: 10.1038/s41598-018-35392-6 (PMC6269487; doi:10.1038/s41598-018-35392-6)
Supplement: Supplementary file 1 — Supplementary Information [file 41598_2018_35392_MOESM1_ESM.pdf]

Supplementary information

Alpha-tubulin acetyltransferase/MEC-17 regulates cancer cell migration and invasion through epithelial–mesenchymal transition suppression and cell polarity disruption

Cheng-Che Lee[1,4], Yun-Ching Cheng[2], Chi-Yen Chang[3], Chi-Min Lin[3], and Jang-Yang Chang[1,3,4*]

1. Center of Infectious Disease and Signaling Research, College of Medicine, National Cheng Kung University, Tainan, Taiwan, ROC
2. Department of Medical Research, Chang Bing Show Chwan Memorial Hospital, Changhua, Taiwan, ROC
3. National Institute of Cancer Research, National Health Research Institutes, Tainan, Taiwan, ROC
4. Division of Hematology and Oncology, Department of Internal Medicine, National Cheng Kung University Hospital, College of Medicine, National Cheng Kung University, Tainan, Taiwan, ROC

*Corresponding author: Jang-Yang Chang, MD
Distinguished Professor and Dean
College of Medicine
National Cheng Kung University
No.1, University Road, Tainan City 701, Taiwan, ROC
E-mail: z10208083@email.ncku.edu.tw or jychang@nhri.org.tw

Supplementary Figure S2 is associated to Figure 1A and 3A.
Supplementary Figure S3 is associated to Figure 4A, 4D and 5B.
Supplementary Figure S4 is associated to Figure 6A-D.
Supplementary Figure S5 is associated to Figure 7A-B.

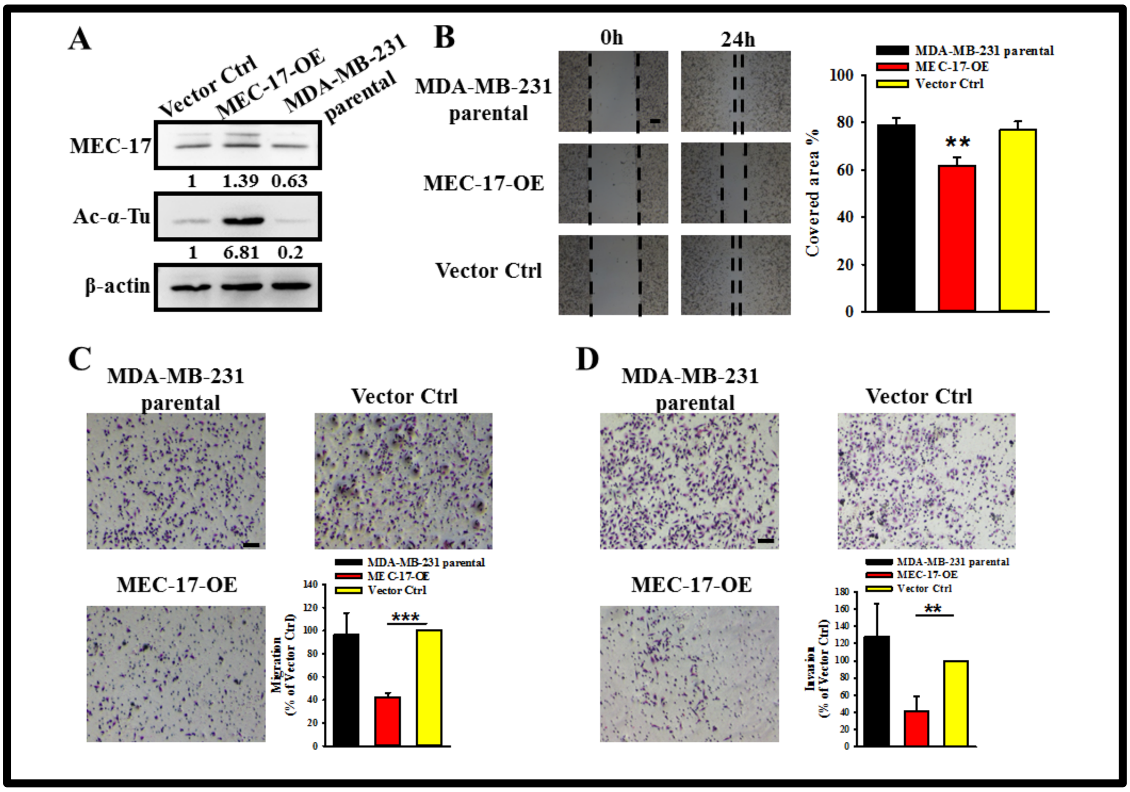

Supplementary Figure S1 Overexpression of MEC-17 in MDA-MB-231 cells attenuates cell migration and invasion. (A) Western blot analysis shows the protein level of MEC-17 and tubulin acetylation in untransduced (MDA-MB-231 parental) and vector control (vector Ctrl) and MEC-17-transduced MDA-MB-231 cells (MEC-17-OE). β-actin served as a loading control. The relative protein intensities were shown. (B) The wound-healing assay showing the cell-covered area of MDA-MB-231 cells untransduced or transduced with vector control or MEC-17-overexpressed constructs after scratching with a pipette tip for 0 and 24 h revealed that MEC-17 attenuates cell migration ability. The representative bar graphs showing the quantification of cell migration ability were displayed by measuring the distance between the front edge of cell movement for each cell condition at 24 h. Scale bar, 100 μm. $^{**}P < 0.01$ in a one-way ANOVA. (C and D) The Transwell assay showing the MDA-MB-231 cell untransduced or transduced with vector control or MEC-17-overexpressed constructs penetrated the lower surface of the filter with or without Matrigel and stained with Giemsa. MEC-17-overexpressed MDA-MB-231 cells exhibited inhibition of migration and invasion. Scale bar, 100 μm. The representative bar graph showing the quantification of migratory or invasive cells compared with the vector control group. $^{***}P < 0.001$, $^{**}P < 0.01$ in a one-way ANOVA.

Fig. 1A

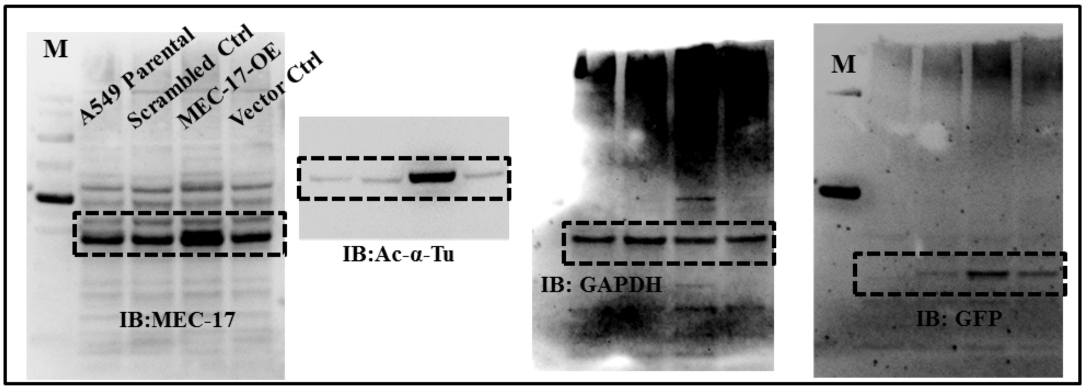

Fig. 3A

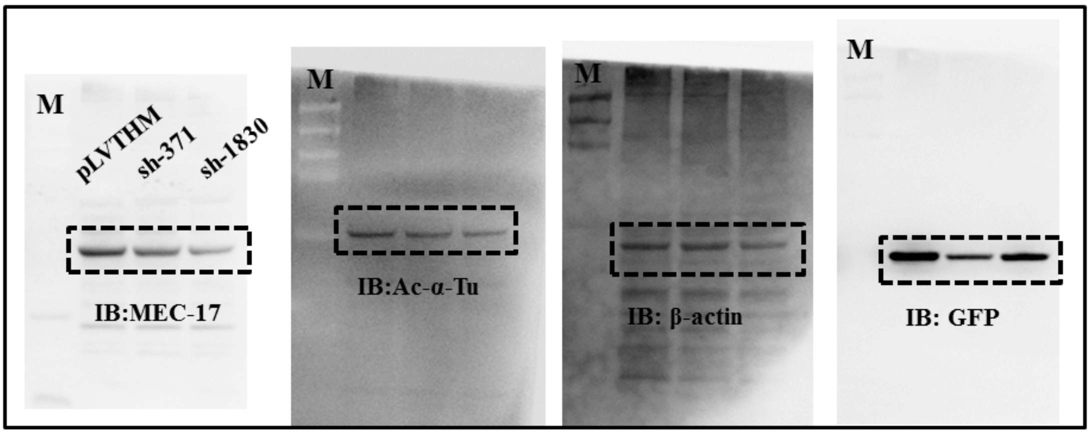

Supplementary Figure S2 Uncropped full-length blots showing the experiments of protein level of MEC-17, Ac-α-Tu, GFP, GAPDH or β-actin presented in the Figure 1A and 3A. Capital letter M represents loading of prestained protein ladder.

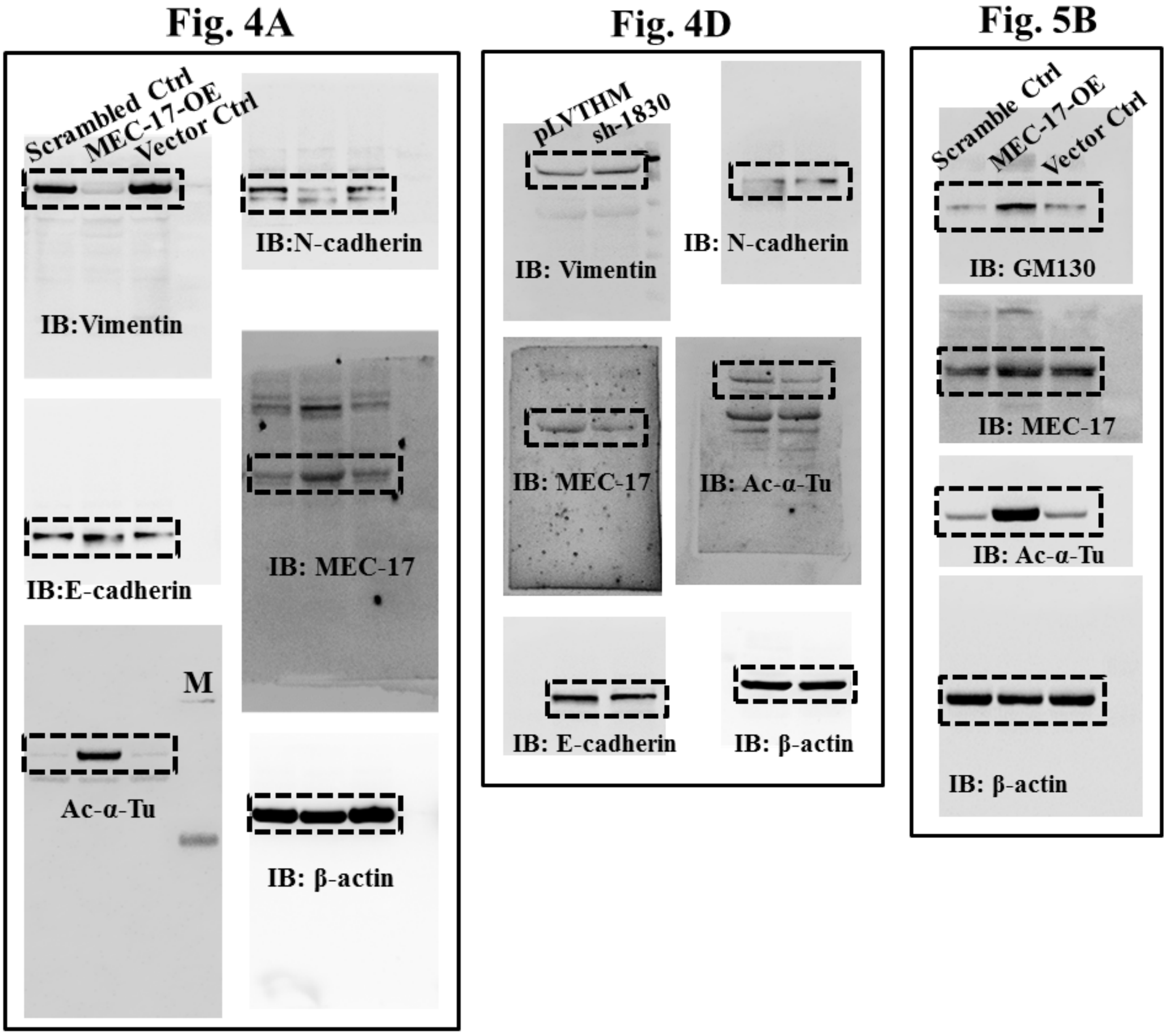

Supplementary Figure S3 Uncropped full-length blots showing the experiments of protein expression of vimentin, N-cadherin, E-cadherin, MEC-17, Ac-α-Tu, β-actin and GM130 presented in the Figure 4A, 4D or 5B. Capital letter M represents loading of prestained protein ladder.

Fig. 6A

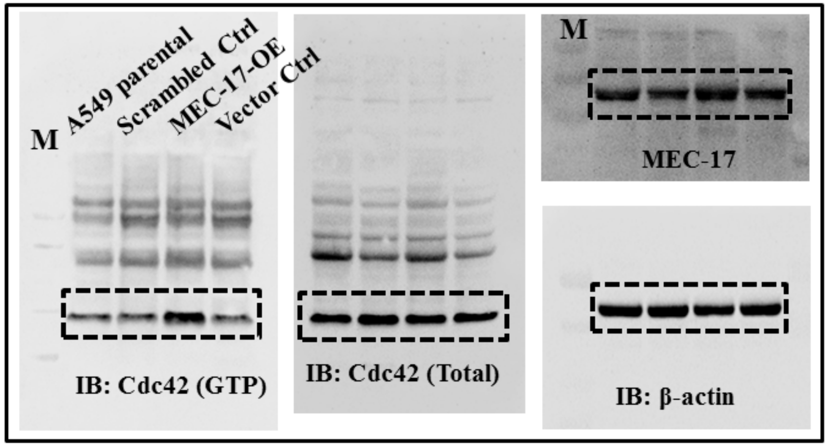

Fig. 6B

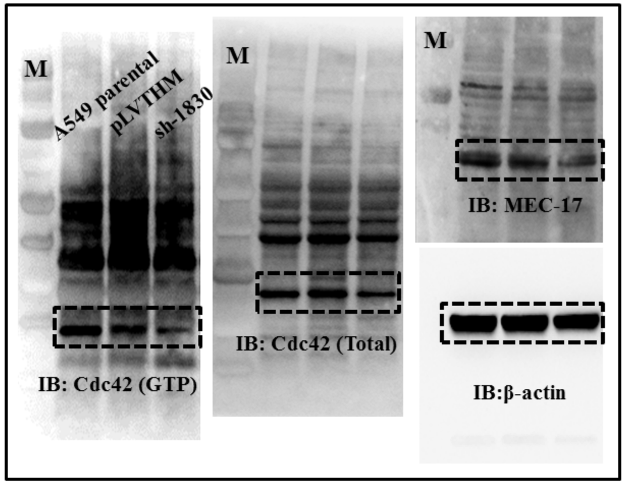

Fig. 6C

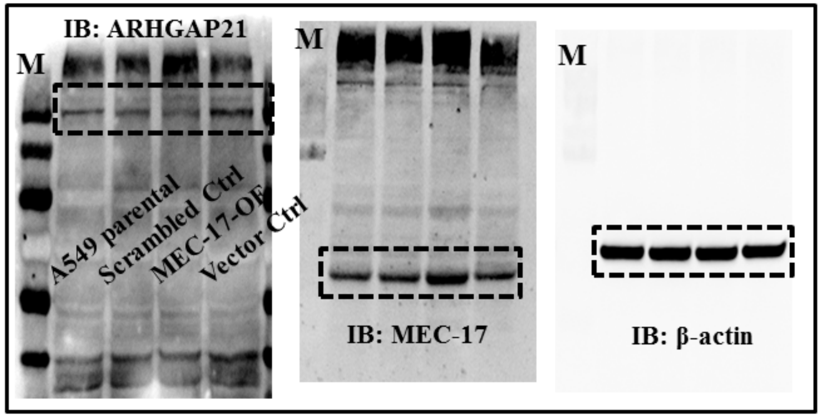

Fig. 6D

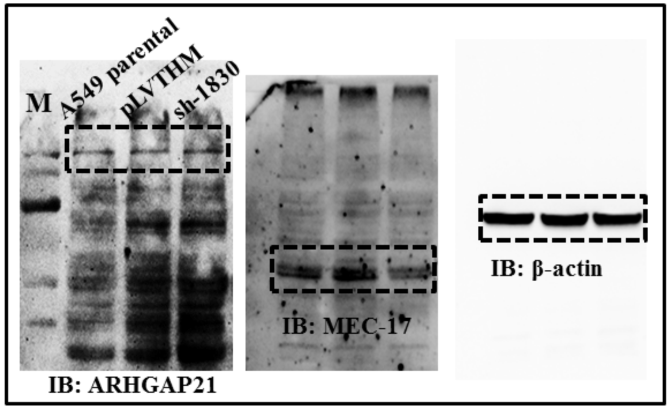

Supplementary Figure S4 Uncropped full-length blots showing the experiments of protein level of cdc42-GTP, total cdc42, MEC-17, β-actin, and ARHGAP21 presented in the Figure 6A-D. Capital letter M represents loading of prestained protein ladder.

Fig. 7A

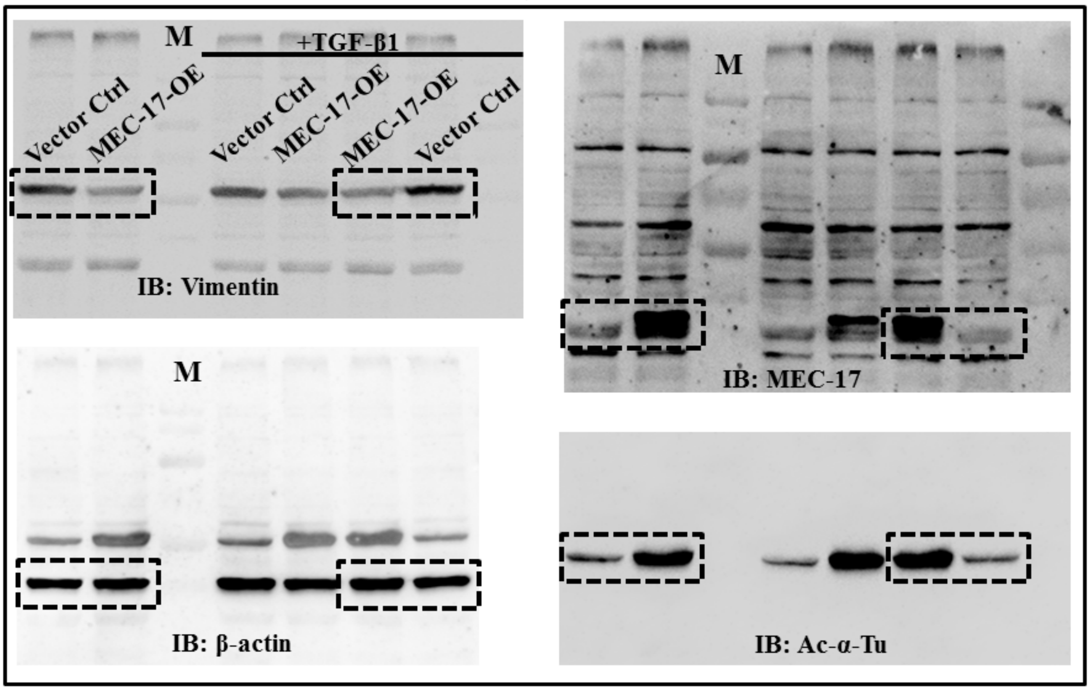

Fig. 7B

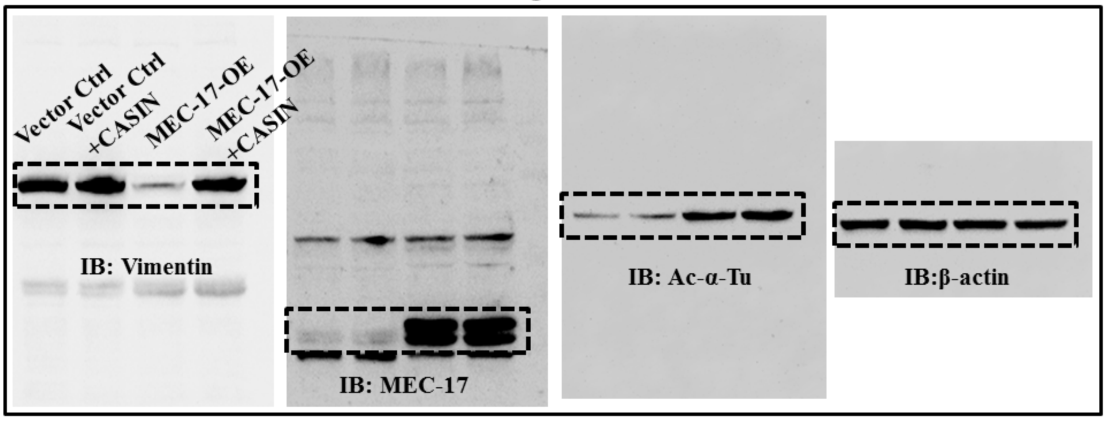

Supplementary Figure S5 Uncropped full-length blots showing the experiments of protein expression of vimentin, MEC-17, Ac-α-Tu and β-actin presented in the Figure 7A-B. Capital letter M represents loading of prestained protein ladder.
